# Immediate Provisionalization and Nonfunctional Loading of a Single Implant in the Maxillary Esthetic Zone: A Clinical Presentation and Parameters for Consideration

**DOI:** 10.1155/2013/378062

**Published:** 2013-12-08

**Authors:** Konstantinos X. Michalakis, Christos D. R. Kalpidis, Yvone Kirmanidou, Hiroshi Hirayama, Pasquale Lino Calvani, Argiris L. Pissiotis

**Affiliations:** ^1^Division of Graduate and Postgraduate Prosthodontics, Tufts University School of Dental Medicine, Boston, MA 02111, USA; ^2^Department of Prosthodontics, Division of Removable Prosthodontics, Faculty of Dentistry, Aristotle University School of Health Sciences, 54124 Thessaloniki, Greece; ^3^Private Practice Limited to Prosthodontics, 54622 Thessaloniki, Greece; ^4^Private Practice Limited to Periodontics, 54623 Thessaloniki, Greece; ^5^Private Practice, 54622 Thessaloniki, Greece; ^6^Private Practice Limited to Prosthodontics, 00198 Rome, Italy

## Abstract

Restoration of single tooth loss with implant supported prosthesis is now considered a highly predictable treatment. However, the maxillary anterior region still presents a challenge for both the prosthodontist and the periodontist because of the inherent difficulties encountered in the provisionalization and harmonic incorporation of the definitive prosthesis into patient's dentogingival complex. This paper presents a clinical case of a single implant placed immediately after the extraction of a maxillary central incisor, followed by immediate provisionalization and nonfunctional loading. The surgical and the restorative techniques are described, and the parameters of consideration for this approach are presented.

## 1. Introduction

Dental implant therapy constitutes a viable treatment modality for both completely [1–4] and partially edentulous patients [5–8]. Furthermore, implants have offered a great service for people, who lost a single tooth due to trauma, carries, internal/external root resorption, or root fracture [9–12]. The biological benefits of not preparing the adjacent teeth for a fixed partial denture fabrication should be emphasized. Conservation of dental tissues, preservation of pulp vitality, respect to periodontium, and maintenance of the residual ridge are the most important factors into consideration.

However, implant treatment had its disadvantages as well. The original protocol, ad modum Brånemark, required an undisturbed healing period of 4 to 6 months, in order for the osseointegration process to take place [3]. Therefore, the provisionalization of a single missing tooth could be a problem, especially in the anterior esthetic area. Originally, removable prostheses were used with negative effects on patient's psychology and an increased risk of force transmissions to the soft tissues overlying the implant. Other alternatives included the fabrication of an etched cast resin-bonded fixed dental prosthesis (Rochette, Maryland or Virginia) [13]. The main advantage of this type of prosthesis is that its fabrication usually requires minimal or no preparation of the abutment teeth. However, high debonding rates [14–16] of these prostheses have been documented, resulting in patient's frustration. Another common disadvantage of both the removable and the etched cast resin-bonded fixed dental prostheses is that their use is very often associated with the loss of the interdental papillae around the definitive implant supported prosthesis [17]. Therefore, the final soft tissue contours of the peri-implant mucosa are far from ideal, jeopardizing the esthetic outcome.

An alternative to the traditional two-stage approach has been presented in the past. This consisted of implant placement and immediate provisionalization. This technique was tested clinically mainly in completely edentulous patients, where cross-arch stabilization was possible, and several studies have reported that the survival rates are similar to those achieved with the unloaded protocol, provided that there was a good bone quality and initial implant stability [18–21]. The “immediate loading” approach was made possible due to changes in the implants' microtopography, achieved with mechanical and/or chemical means [22–26]. This had as a result a faster induction of bone formation onto the alloplastic surface of dental implants, as shown in several animal models and histomorphometric studies in humans [27–29].

This new concept of immediate provisionalization was also applied to single implants, with very good results [30, 31]. It should be emphasized however that, a very careful case selection is essential in order to have a predictable biologic, functional and esthetic outcome.

This paper presents a clinical case involving the extraction of a root fractured maxillary central incisor and immediate placement of an implant, followed by immediate provisionalization and non-functional loading.

## 2. Case Description

**Figure 1:**
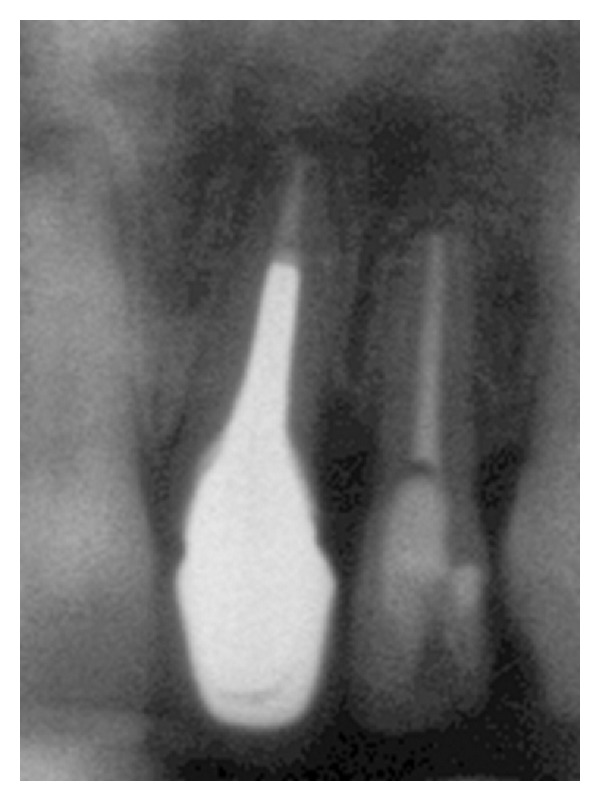
Initial radiograph of maxillary left central incisor.

**Figure 2:**
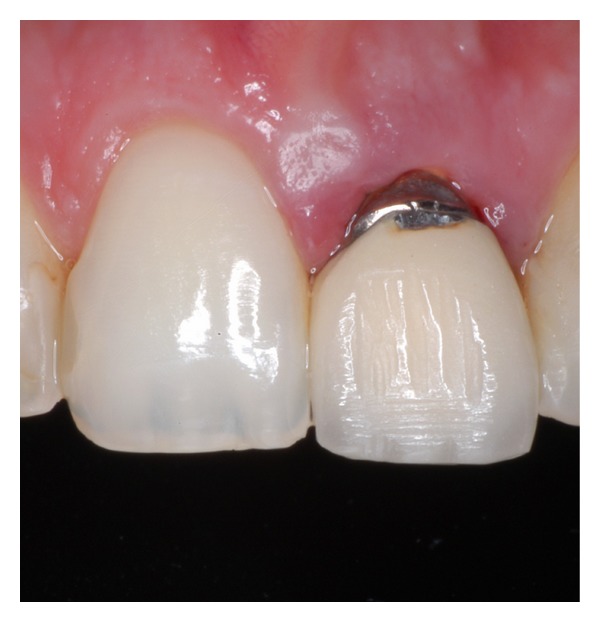
The maxillary left central incisor with a metal ceramic restoration. Its margins were placed on the underlying cast post and core.

A 25-year-old healthy Caucasian woman presented with a metal ceramic restoration on the left maxillary central incisor, that did not satisfy her esthetic demands anymore. In addition, the patient was feeling a dull pain in the area. The patient did not smoke and was healthy. The radiographic examination showed an oversized cast post and core, which occupied three quarters of the root's length (Figure 1). The clinical examination revealed that the metal ceramic restoration was fractured at the buccal marginal area, it had a surface texture that did not match that of the adjacent natural teeth, it was rather opaque, and its margins were placed on the underlying cast post and core instead of on sound tooth structure (Figure 2). Soft tissue inflammation was evident around the crown's marginal area. An 8 mm pocket depth was found at the distal area of the tooth. The examination also demonstrated a discrepancy of the soft tissue contours between the two central incisors, sufficient width of keratinized tissue in the maxillary anterior area and a scalloped thin periodontium [32–34].

Treatment options were presented to the patient after assessment of all clinical and radiographic data. The patient wished to restore the failing tooth with an implant-supported restoration.

Prior to implant surgery, preliminary impressions were obtained by using irreversible hydrocolloid impression material. Diagnostic casts for both the maxillary and mandibular arches were fabricated with type III dental stone and they were mounted in maximal intercuspal position on a semiadjustable articulator. A diagnostic waxing was made and the maxillary cast was duplicated. A surgical stent was then fabricated with clear autopolymerizing polymethyl-methacrylate resin. The tooth's cingulum area was marked and an access channel was opened with a 261E-023 laboratory carbide bur (Brasseler, USA). A provisional restoration was also fabricated by using autopolymerizing acrylic resin (GC Unifast III, GC Tokyo, Japan). Two acrylic resin retainers resting on the palatal surfaces of the adjacent teeth were incorporated into the provisional restoration, in order to assist to the orientation of the provisional crown during the surgery. Finally, the provisional restoration was hollowed out and an access hole was opened palatally.

**Figure 3:**
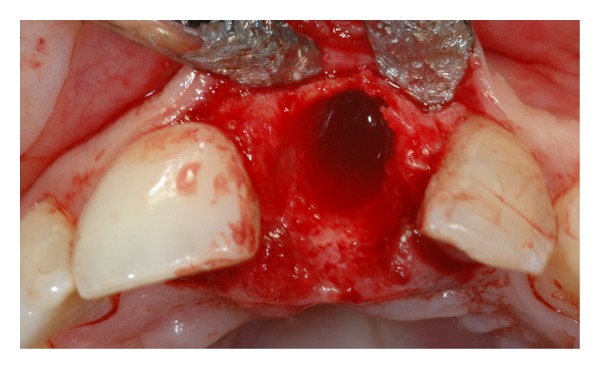
Atraumatic extraction of the maxillary left central incisor.

**Figure 4:**
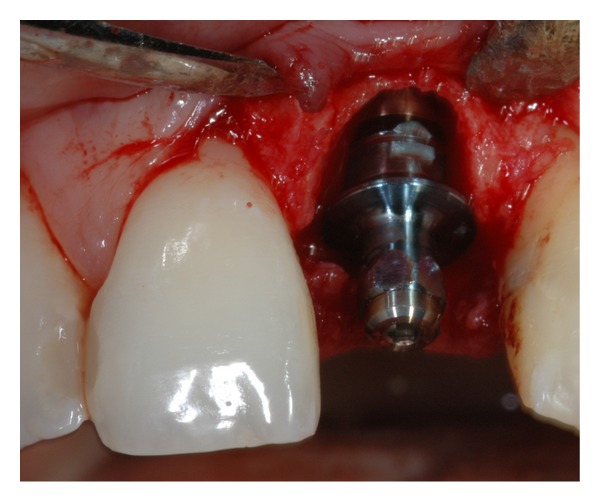
Placement of an Osseotite NT implant in the extraction socket. The platform of the implant was placed 2 mm apically to the cementoenamel junction of the right maxillary central incisor.

On the day of the surgery, local anesthesia with 4% articaine (1 : 100,000 epinephrine) (Ubistesin Forte, 3M/ESPE) was delivered and a full thickness flap was raised. An atraumatic tooth extraction was then performed with the use of a periotome, taking extra care to preserve the buccal plate (Figure 3). The osteotomy started with a round bur which was placed into the channel of the surgical stent previously described, and progressed with the 2.3 mm twist drill, the pilot drill, and the 3.25 × 13 mm shaping drill at 500 rpm, with the drill torque test set at 50 Ncm. Attention was given to engage with the drills the palatal wall of the extraction socket. The osteotomy was performed under copious saline irrigation. A 4 × 13 externally hexed Osseotite NT implant (Biomet 3i, Palm Beach Gardens, FL) was placed using 20 rpm at 40 Ncm torque. The platform of the implant was placed 2 mm apically to the cementoenamel junction of the right maxillary central incisor (Figure 4) [35–37].

A temporary metal cylinder was fastened with a titanium screw on the implant and marked in order to be reduced to the proper length. It was then removed, fastened to an implant replica, and cut with a silicon separating disc. It was further reduced and shaped with diamond burs. The final length and shape of the temporary cylinder was checked intraorally, with the provisional restoration in place. Furthermore, the clearance between the temporary cylinder and the provisional restoration was verified. Autopolymerizing polymethyl-methacrylate acrylic resin (Jet, Lang Dental, Wheeling, IL) was added intraorally between the provisional restoration and the temporary cylinder, using the bead-brush technique. After the polymerization was completed, the provisional restoration-temporary cylinder complex was removed and fastened on an implant replica. More autopolymerizing polymethyl-methacrylate acrylic resin was added to support the adjacent soft tissues and the restoration was placed in a pressure pot containing warm water. It was then shaped with laboratory carbide burs and polished with abrasive rubber points (Bredent, Senden, Germany) and pumice. The provisional restoration was fastened on the implant with a torque driver (Anthogyr, Sallanches, France) with a 20 Ncm torque. The palatal access hole was closed with gutta-percha and acrylic resin. Occlusion was checked and any centric and eccentric contacts were eliminated. The acrylic was further polished with abrasive points.

**Figure 5:**
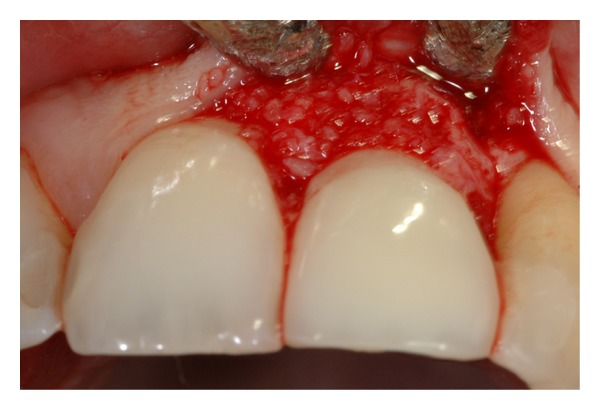
Buccal bone deficiency filled with bovine bone material.

**Figure 6:**
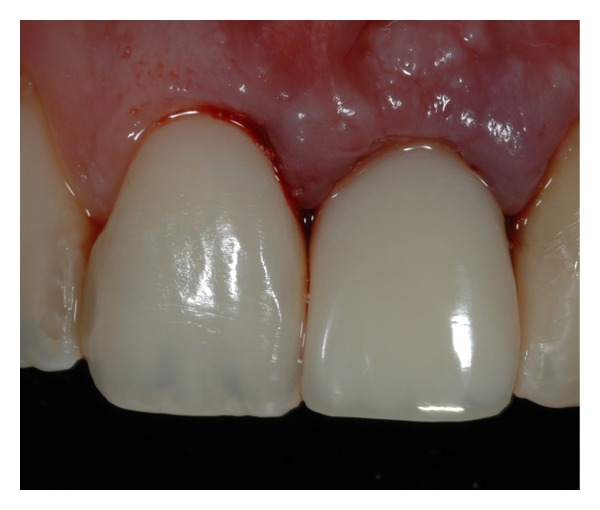
Initial provisional PMMA acrylic resin crown.

A bone deficiency remained buccally and it was filled with bovine bone mineral (Bio-Oss Collagen, Geistlich) (Figure 5). The flap was sutured in place. Postoperatively, the patient was prescribed 1 g of amoxicillin/clavulanate potassium (Augmentin; GlaxoSmithKline) per day for 6 days and the nonsteroidal analgesic nimesulide (Mesulid; Boehringer Ingelheim) (100 mg, twice a day for 6 days). A 0.2% chlorhexidine (Chlorhexil; Intermed, Greece) rinse was also prescribed and the patient was instructed to use it three times a day for 14 days. The sutures were removed after 10 days (Figure 6). Patient was placed on a soft food diet for 6 weeks.

**Figure 7:**
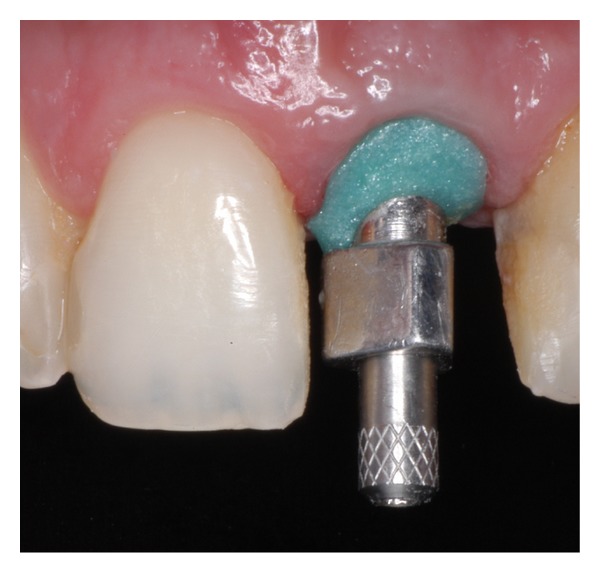
The impression coping was modified with light-polymerizing resin material to register the peri-implant soft tissues.

**Figure 8:**
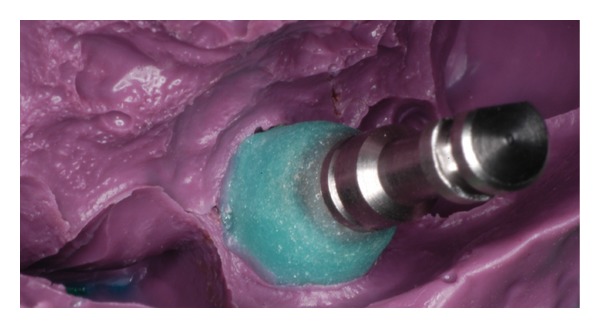
The impression coping into the polyether impression material used. Note the amount of light-polymerizing resin material added to support the soft tissues.

**Figure 9:**
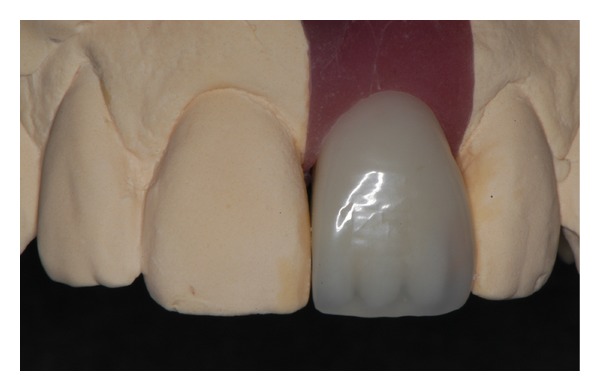
A new provisional crown was fabricated with light-polymerizing resin, in order to better support the soft tissue contours mesially and provide a better esthetic result.

**Figure 10:**
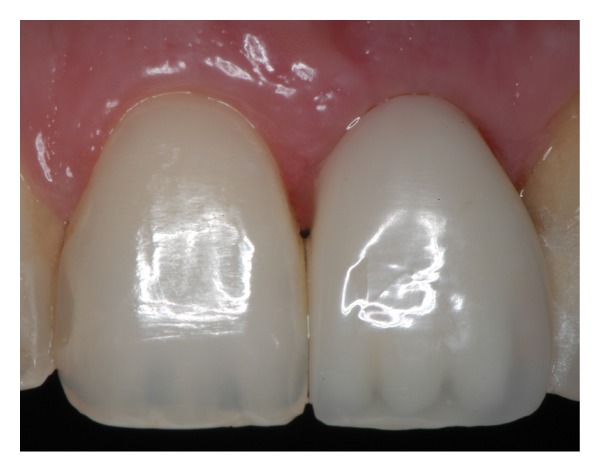
Clinical view of the new provisional crown.

Two months after implant placement, the provisional restoration was removed and an impression coping was screwed onto the top of the implant. Light-polymerizing resin material (Liquidam; Discus Dental, Culver City, CA) was injected around the impression coping and was immediately polymerized with a UV polymerizing unit, preventing the soft tissues from collapsing onto the impression coping (Figure 7). A plastic stock tray and polyether material (Impregum, 3M/ESPE, Seefeld, Germany) were then used for the impression (Figure 8). A new screw-type provisional restoration was fabricated by using a temporary cylinder and light-polymerizing resin (Gradia; GC Tokyo, Japan) (Figure 9). This provisional restoration was fabricated in order to support better the soft tissue contours mesially and provide a better esthetic result (Figure 10). No centric or eccentric contact was given to the crown.

**Figure 11:**
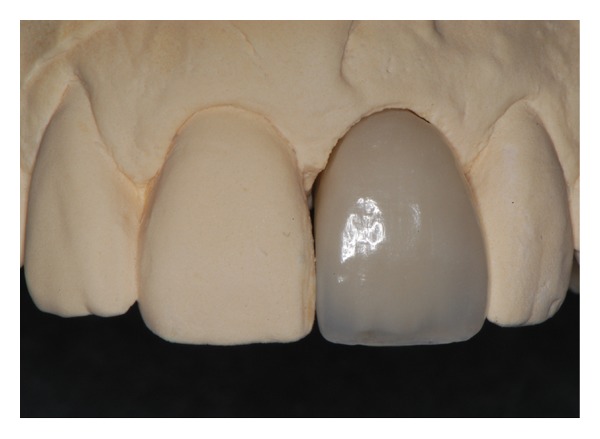
A definitive screw retained metal ceramic restoration was fabricated 6 months after implant placement.

Regular examinations were performed at 3-week intervals for oral hygiene and occlusion evaluation. After 6 months of uneventful healing, a periapical radiograph was taken, showing no areas of radiolucency around the implant. The provisional restoration was removed and a definitive impression was obtained with the method previously described. A definitive cast was fabricated from type IV dental stone and mounted in maximal intercuspal position with the mandibular cast on a semiadjustable articulator. A UCLA abutment with a gold hexed collar was used for the definitive restoration. This was fastened onto the implant replica and waxed to full contour. A silicone key was made and the wax-up was then cut back, invested and cast with high noble alloy. Porcelain was applied and baked in the traditional manner (Figure 11). The restoration was tried-in, and proximal contacts and occlusion were adjusted. After patient's approval, the restoration was returned to the laboratory for the glazing procedure.

**Figure 12:**
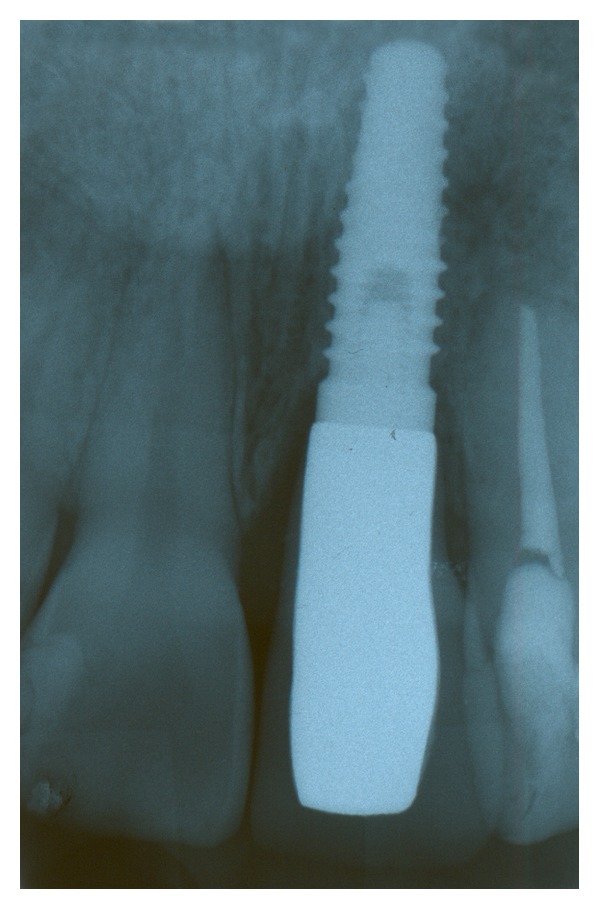
Final radiograph obtained at 6 months after implant placement.

**Figure 13:**
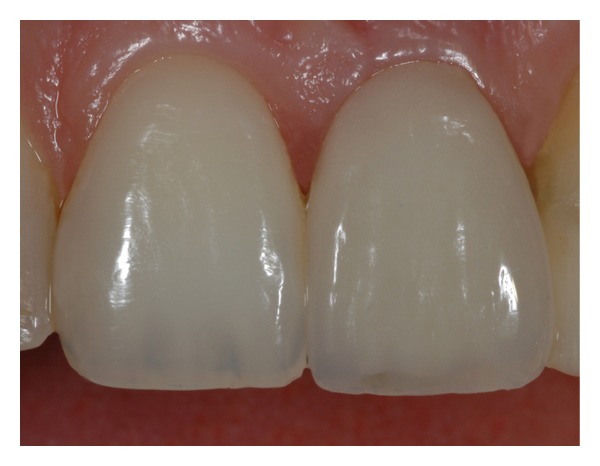
Clinical photograph of the definitive metal ceramic crown.

After the delivery of the definitive restoration (Figures 12 and 13), the patient was followed every 4 months for the next year. She was then placed on a 6-month recall basis. Soft tissues around the implant supported restoration appeared stable with no signs of inflammation or recession. The interdental papillae looked normal enhancing the optimal esthetic result obtained by the definitive metal ceramic restoration.

## 3. Discussion

The present report demonstrated a successful use of an immediate provisionalization and non-functional loading of a single implant in the maxillary esthetic zone. This approach had a positive psychological impact on this young female patient, as it immediately improved her appearance and confidence. This treatment approach was also based on preliminary data regarding the maintenance of the residual ridge in both height and width and the support of peri-implant tissues with the provisional restoration, including the preservation of the interdental papillae around the definitive restoration [38–40]. It should be mentioned, however, that recent animal histologic research has demonstrated that immediate implant placement cannot prevent bone resorption, which is a physiologic metabolic process after tooth extraction [41, 42]. This has also been confirmed from studies in humans, showing also that the bone resorption of the buccal plate is almost double when compared to that of the palatal or lingual plate [43]. Therefore, in the immediate placement approach the engagement of the palatal wall, which is crucial for implant's initial stability, always leaves a space between the implant and the coronal part of the buccal plate. Clinical studies indicate that in spaces with widths up to 2 mm, barrier membranes or regenerative techniques are not needed [44, 45]. This finding justifies the use of bovine bone mineral material without any barrier membrane for the treatment of the buccal bone deficiency after implant placement in the patient presented.

The Osseotite NT implant that was selected for the treatment of the patient presents a greater thread pitch (0.9 mm versus 0.6 mm for cylindric implants) and has the advantage of a taper form which mimics the natural form of the tooth, making it ideal for fresh extraction sites [46–48]. The surface of this type of implants receives a chemical thermal etching with a combination of hydrochloric and sulfuric acids, in order to induce a faster bone formation onto it. Histological and 3-year clinical data from multicenter studies confirm that high success rates can be obtained by its use [49–52].

The implant was placed in a more apical position in order to provide adequate space for the harmonic emergence of the prosthetic reconstruction through the soft tissues and its gradual transition from the size of the implant's platform to that of the emergence profile of natural anterior teeth [53]. It should be noted that the greater the difference between the diameter of the implant and the size of the tooth to be restored, the more apical the position of the implant should be in order to provide the appropriate space for the natural emergence of the restoration [36]. A more coronal implant placement may jeopardize the final esthetic result, since the abrupt transition from implant's platform to the restoration's normal contour will have as a result the exertion of big pressure on the surrounding soft tissues [36, 53]. This fact in conjunction with the establishment of the biologic width may ultimately lead to a soft tissue gradual recession and disclosure of the prosthetic components or even of the implant's coronal part. Conversely, if the implant is placed too apically, the respective migration of the microgap signals a bone recession, which is also observed in 1-piece implants [54]. This may cause a soft tissue recession, especially when the bone loss is accompanied by a thin buccal plate [36, 37]. In the patient presented, the cementoenamel junction of the maxillary right central incisor has been used as a reference point for the determination of implant's placement in the apico-coronal direction. In a healthy periodontium, the placement of the implant's platform 2 mm more apically than the cementoenamel junction of the adjacent teeth can be a good starting point [35–37]. It has also been suggested that, for patients with a thin periodontic biotype, the implant's platform should be placed 3-4 mm more apically than the adjacent bone peak [53, 55].

**Figure 14:**
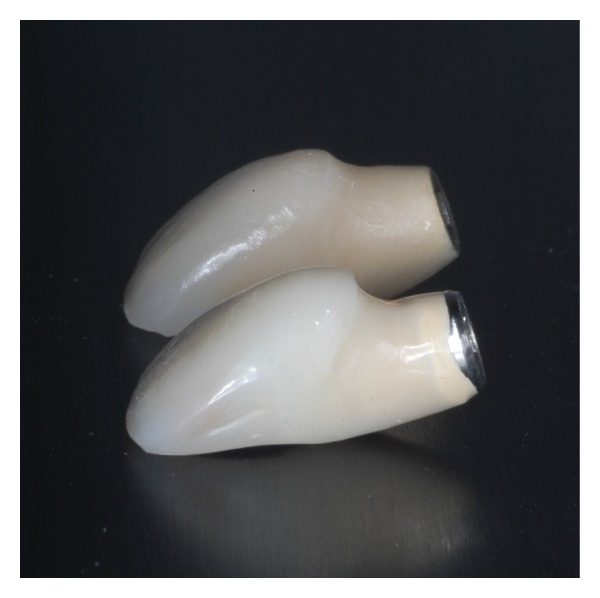
The ridge lap observed at the second provisional crown (down) was modified at the definitive crown (up) to facilitate better oral hygiene.

Regarding the labio-palatal dimension, the osteotomy was made after engaging the intact palatal wall of the socket, in order to obtain initial implant stability and to keep a safe distance from the defected and sensitive buccal wall [53, 56]. It should be mentioned, however, that the more palatal the implant is placed, the more space is needed on the vertical axis for a smooth and harmonic emergence of the restoration. Placing this concept in numbers, it can be said that for every 1 mm of palatal displacement, the implant should be placed 1 mm more apically [36, 57]. Nevertheless, a problem to the restoration's natural emergence can be created when the implant is placed too far palatally. In those cases, a restoration with a ridge lap is usually made in order to achieve a natural tooth appearance. However, this approach has negative consequences in dental hygiene [36, 37, 58, 59]. The ridge lap provisional restoration which was fabricated two months after the implant placement was modified at the definitive crown in order to facilitate better access for effective hygiene measures (Figure 14). The decision for a new provisional restoration fabrication was based on clinical data suggesting that a successful functional loading can be obtained two months after implant placement [60, 61].

The clinicians have many restorative options for the implant supported single crown. A screw retained prosthesis was selected for the provisional stage because of the ideal implant placement in relation to the natural contours of the maxillary incisor. Additionally, a cement-retained prosthesis could possibly jeopardize the healing process due to possible cement residues which can cause peri-implant inflammation [62].

A metal-ceramic restoration was the definitive restoration of choice since this was tested during the provisionalization period with no adverse esthetic results. Clinical studies have demonstrated that when the mucosal thickness is greater than 3 mm, no difference between all- and metal-ceramic restorations can be detected by the human eye [63].

The case presented consisted of a maxillary central incisor tooth extraction and immediate non-functional implant loading. This approach preserved both soft and hard tissues, and created a harmonious relationship among the implant, the restoration and the surrounding tissues. What should be emphasized is the necessity of the appropriate case evaluation and selection as well as the careful treatment planning. These parameters along with patient's compliance are of utmost importance for a successful outcome.
